# Intra-tester and inter-tester reliability of maximum isokinetic hip and knee strength measurements with the exoskeleton in healthy young adults

**DOI:** 10.3389/fspor.2025.1696954

**Published:** 2026-01-09

**Authors:** Jiang-Li Zhao, Hao Xie, Pei-Ming Chen, Shamay S. M. Ng, Chu-Huai Wang

**Affiliations:** 1 Department of Rehabilitation Medicine, The First Affiliated Hospital, Sun Yat-sen University, Guangzhou, China; 2 Department of Rehabilitation Sciences, The Hong Kong Polytechnic University, Hong Kong SAR China

**Keywords:** dynamometry, lower extremity, reliability, strength, test, wearable robotics

## Abstract

**Purpose:**

The UGO exoskeleton enables gait training and simultaneous, *in-situ* assessment of peak isokinetic torque at the hip and knee joints while participants maintain an upright, near-natural walking posture. The primary aim of this study was to establish the inter- and intra-tester reliability of peak isokinetic torque using the UGO exoskeleton in healthy young adults.

**Methods:**

Two independent cohorts were enrolled to determine the inter- and intra-tester reliability of the UGO exoskeleton. Each cohort consisted of 20 fourth-year undergraduate students (10 male, 10 female), with mean ages of 22.55 ± 1.05 and 21.40 ± 1.00 years, respectively. Testing was conducted by two experienced therapists. Peak isokinetic torque (Nm) in hip and knee flexors and extensors was collected. Relative and absolute reliability of peak torques were estimated using the intra-class correlation coefficient (ICC) and standard error of measurement (SEM), respectively. Bland–Altman plot was utilized to further elucidate the differences between tests obtained by different testers or by the same tester at different times.

**Results:**

Inter-tester reliability was good-to-excellent, as evidenced by ICCs of 0.791–0.906 for the hip and 0.859–0.975 for the knee (all *P* ≤ 0.001). Intra-tester reliability was excellent, with ICCs of 0.834–0.905 for the hip and 0.917–0.955 for the knee (all *P* < 0.001). The SEM values further indicated moderate to high reliability, spanning 13.1–16.1 Nm (11.9–15.6%) at the hip and 8.5–10.2 Nm (8.8–16.0%) at the knee for inter-tester assessment, and 11.5–13.1 Nm (10.2–13.1%) at the hip and 6.4–8.0 Nm (8.2%–9.2%) at the knee for intra-tester assessment. Bland-Altman plots confirmed this, showing mean differences from −3.3 to −26.5 Nm (inter-tester) and −1.4 to −15.7 Nm (intra-tester), and 95% limits of agreement ranging from −71.2 to 31.2 Nm and from −51.8 to 26.2 Nm, respectively.

**Conclusions:**

The UGO exoskeleton provides a reliable, time-efficient, and functionally relevant means of quantifying hip- and knee-flexor and -extensor torque in healthy young adults while maintaining a posture that closely replicates natural gait.

## 1 Introduction

Muscle strength assessment is a cornerstone of modern clinical and sports practice because it serves as an objective biomarker of neuromuscular integrity, functional capacity, and recovery trajectory (1). Accurate strength quantification supports the early identification of pathological weakness caused by neurological disorders (e.g., stroke and spinal cord injury) and musculoskeletal conditions (e.g., tendinopathies and sarcopenia), enabling timely diagnosis and appropriately targeted interventions (2, 3). Serial strength measurements are a sensitive marker of rehabilitation progress; even small changes in torque output can indicate neural adaptation, muscle hypertrophy, or therapeutic efficacy, thereby guiding clinicians in adjusting exercise dosages and training modalities (4, 5). Standardised strength data obtained through isokinetic or isometric dynamometry, neuromuscular control tests such as functional hop tests (6), and functional performance measures such as the Star Excursion Balance Test (7) support evidence-based return-to-play or return-to-work decisions by confirming that injured individuals have regained their pre-morbid capacity, ultimately improving quality of life and reducing the costs of re-injury (8, 9). Thus, reliable muscle strength evaluation links laboratory-derived metrics with real-world function and supports both clinical precision and performance enhancement (10).

Isokinetic dynamometry has long been regarded as the gold standard for evaluating isolated joint torque output because it allows control of angular velocity and provides objective and reproducible data (11, 12). However, traditional isokinetic devices (e.g., Biodex and Cybex) are limited by complex operating procedures and a large physical footprint, which restrict portability and widespread clinical adoption. These systems also require time-consuming, technician-dependent setup that increases inter-rater variability (13). Traditionally, isokinetic testing has been conducted in seated or supine positions (e.g., for hip or knee movements). Although these positions effectively isolate prime mover muscles and have high reliability, they assess strength in a non-weight-bearing and non-functional context that does not represent real-world athletic or daily activities. Moreover, most of these systems restrict assessment to open-chain, single-plane movements performed at constant velocities, which do not reflect the multi-joint and variable-speed demands of real-world functional tasks (14). Although some equipment, such as the IsoMed 2000, has been developed for closed-chain, multi-joint isokinetic assessment, it has systematic limitations in angular velocity control, posture standardisation, and height-related bias, which may affect its accuracy in measuring muscle strength (15). Hand-held dynamometry (HHD) is also used to assess muscle strength. The device is set up or held by the therapist to obtain an objective measurement of force. HHD is valued for its portability, low cost, and ability to provide objective and quantitative strength data in almost any clinical setting, making it especially useful for serial bedside assessments and resource-limited environments (16). A previous study (17) reported moderate-to-excellent intra-rater reliability for HHD [intraclass correlation coefficient (ICC) = 0.66–0.91] with a minimal detectable change (MDC) of 27.5–52.4 Nm in assessing knee and hip flexor and extensor strength in healthy individuals. However, the utility of HHD is constrained by the lack of universally standardised testing positions (18). Strong muscles may be underestimated when the clinician is unable to hold the HHD firmly enough to counter the participant's force, particularly when the participant exerts a force greater than the clinician can resist (19). These limitations introduce inter-tester variability and ceiling effects in stronger individuals, compromising measurement reliability and validity (20). Moreover, most HHD devices measure isometric strength instead of isokinetic strength, which limits their use in evaluating dynamic muscle performance at controlled velocities.

Robotic exoskeletons have emerged as promising alternatives because they integrate real-time feedback and automated control to standardise testing procedures (21, 22). The powered UGO lower-limb exoskeleton was developed to support balanced walking training for people with stroke or spinal cord injury as early as possible (23). This exoskeleton stabilises the trunk, pelvis, hip, knee, and ankle joints, allowing users to walk safely with correct posture. In addition to its role in rehabilitation training, the UGO exoskeleton may also address the limitations of traditional isokinetic devices by eliminating the need for additional, costly, purpose-built assessment equipment, thereby providing both economic and practical benefits. Its mechanical stability ensures objective, evaluator-independent measurements, thus enhancing reliability and consistency in strength assessment. Furthermore, the standing posture, which is the closest functional posture for walking, is used for muscle strength assessment in the UGO exoskeleton. An isokinetic test of hip and knee extension performed in an upright, single-leg stance can more accurately simulate the eccentric (braking) and concentric (propulsive) demands of walking than a seated test. The upright position is the foundation of most human movement, including walking, running, jumping, and throwing. Testing in this posture allows strength and power to be evaluated in a kinetic chain context. By contextualising strength assessment within the functional kinetic chain, this approach provides clinicians and researchers with a powerful tool for profiling athletic performance, designing targeted rehabilitation programmes, and making informed, data-driven return-to-sport decisions that match the actual demands of the activity. This testing method has effectively transformed the dynamometer from a simple strength-measuring device into a comprehensive neuromuscular evaluation system. Additionally, the method of band fixation used during muscle strength assessment is the same as the method used during walking training. This consistency enables the rapid assessment of muscle strength before and after training without requiring additional assessment equipment or procedures. Although the UGO exoskeleton offers these benefits, few studies have comprehensively validated its reliability for hip and knee isokinetic assessment in healthy and clinical populations.

The assessment of inter- and intra-tester reliability is fundamental to this study because the validity of any quantitative biomechanical measurement depends on a consistent and reproducible measurement protocol. For the UGO exoskeleton to function as a credible tool in clinical and research settings, its output must be consistent and independent of the operator. This evaluation demonstrates that the multi-step participant setup process, which requires precise alignment and secure strapping, can be performed reproducibly. High inter-tester reliability ensures consistent results across different clinicians and therefore supports generalised application, while high intra-tester reliability confirms measurement stability over time, which is essential for accurately tracking patient progress. By quantifying both inter-tester and intra-tester reliability, we have validated the entire testing protocol and provided future users with the error estimates needed to distinguish meaningful clinical change from measurement noise. This information supports the confident adoption of this device in personalised rehabilitation and multi-centre trials. Specifically, in this study, we investigated the intra- and inter-tester reliability of the UGO rehabilitation exoskeleton in measuring maximum isokinetic hip and knee strength in healthy young adults.

## 2 Materials and methods

### 2.1 Study design

This study used a cross-sectional design to investigate the reliability of peak isokinetic torque measurements in four muscle groups, namely hip flexors, hip extensors, knee flexors, and knee extensors, measured bilaterally in fourth-year undergraduate students. The UGO exoskeleton was used as the testing apparatus.

### 2.2 Study setting

This study was conducted in the Department of Rehabilitation Medicine of the First Affiliated Hospital of Sun Yat-sen University, China.

### 2.3 Participants

Participants were recruited through posters displayed for fourth-year undergraduate students completing an internship in the Department of Rehabilitation Medicine at the First Affiliated Hospital of Sun Yat-sen University between January and July 2022. All participants reported having no specialised training relevant to the study protocol or previous experience using the device. The inclusion criteria were as follows: (i) willingness to participate in the experiment and ability to provide informed consent; (ii) availability to complete two testing sessions within 3 days; (iii) no history of fractures or neuromuscular injuries in both lower limbs and no lower-back pain that could affect lower-limb muscle strength; (iv) no history of central nervous system damage that could affect motor function or muscle strength; (v) no recent use of medications that might affect muscle strength; (vi) no other reported medical conditions such as acute infection or cardiovascular disease; and (vii) right-leg dominance (leg dominance was determined by asking each participant to kick a ball placed directly in front of them; use of the right leg indicated right-leg dominance.).

### 2.4 Ethics

This study was approved by the Human Subjects Ethics Subcommittee of the First Affiliated Hospital of Sun Yat-sen University in China [ethical approval number: (2020)430]. Written informed consent was obtained from all participants. This study was conducted in accordance with the Declaration of Helsinki.

### 2.5 Exoskeleton

This study evaluated the reliability of peak isokinetic torque measurements obtained using a commercial lower-limb exoskeleton (UGO 220, Hangzhou RoboCT Technology Development Co., Ltd). The rehabilitation exoskeleton uses sophisticated sensor technology for precise data acquisition. Each motor-driven joint is equipped with a magnetic rotary encoder (AS5048A, AMS, Inc.) that provides 14-bit resolution for accurate joint angle measurements and a customised torque sensor for real-time torque recording. Hip and knee flexion and extension are driven by servo motors paired with harmonic reducers capable of generating up to 100 Nm. The customised torque sensor has a measurement range of 150 Nm, a resolution of 0.05 Nm, and an accuracy of 0.3% full scale. Both angle and torque signals are recorded at a sampling rate of 100 Hz (23).

### 2.6 Testing

**Figure 1:**
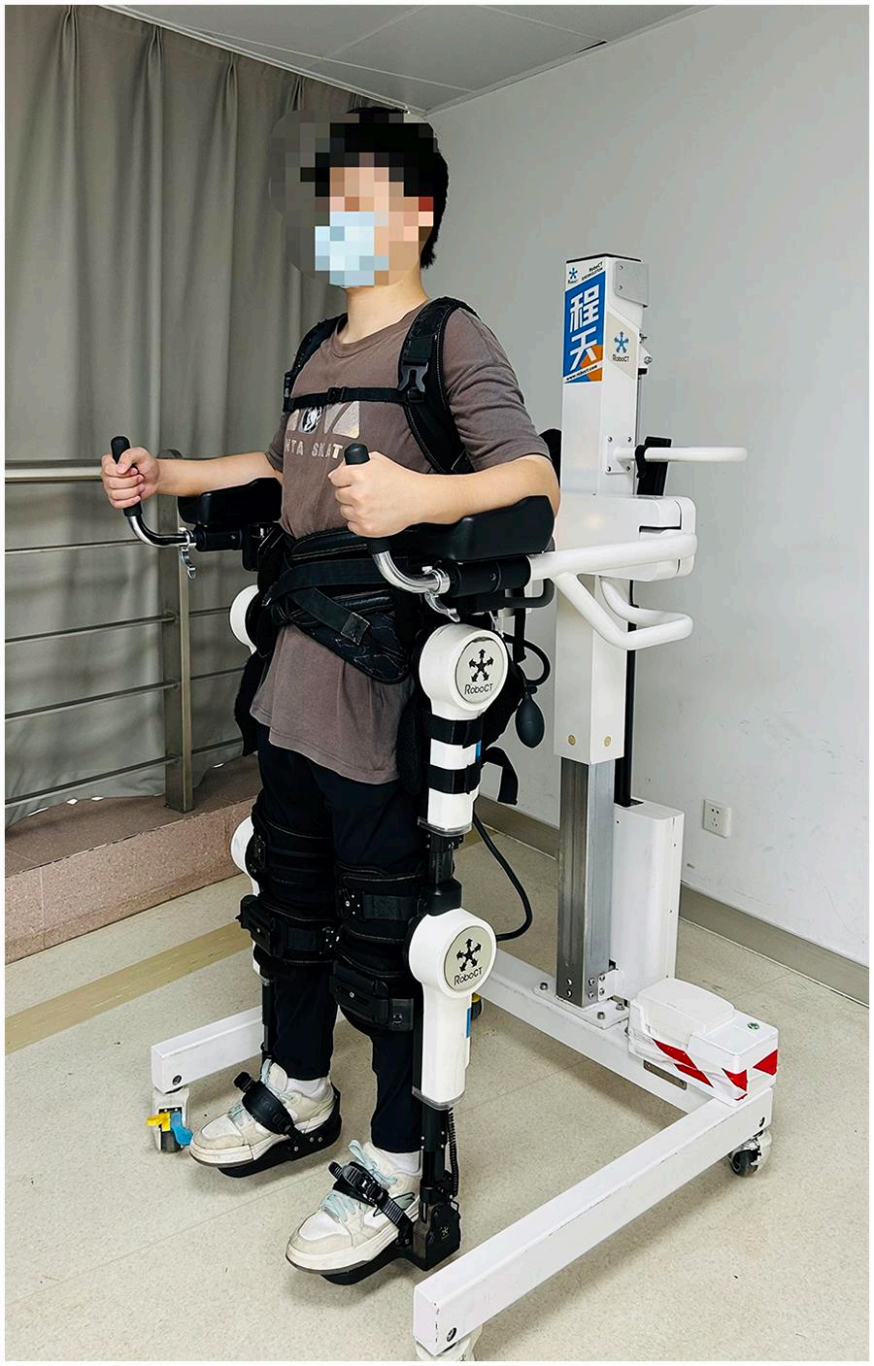
Participant positioning and limb stabilisation for the isokinetic strength test.

The posture and fixation used for testing are illustrated in Figure 1. Participant fixation was conducted as follows. With the participant seated firmly against the robot's backrest, the operator first confirmed alignment between the robot's hip-joint axis and the participant's greater trochanter. The lengths of the robotic thigh and calf segments were then adjusted using the control panel to align the robot's knee-joint axis with the anatomical axis of the participant's knee. The trunk was subsequently stabilised using three abdominal belts, and straps were applied to secure the thigh, calf, and foot. All straps were tightened to a snug fit, defined as allowing the comfortable insertion of one finger. The robot was then moved from the seated to the standing position using the remote control. After the transition, the trunk straps were re-fastened, and the lateral width of the robot was adjusted to limit movement of the pelvis in the coronal plane. These steps ensured that the equipment could be fitted appropriately to participants of different heights and weights.

Peak torque was assessed bilaterally in four muscle groups, namely hip flexors, hip extensors, knee flexors, and knee extensors, using the UGO exoskeleton. The testing sequence for each participant was the dominant hip, non-dominant hip, dominant knee, and non-dominant knee.

The UGO exoskeleton was preset to have consistent angular velocities and ranges of motion for all tests. The angular velocity for both the hip and knee joints was 60°/s. The range of motion was −10° to 60° for the hip joint and 0° to 70° for the knee joint.

Maximum isokinetic strength tests were administered by two therapists (ZJ and XH) who were familiar with the UGO exoskeleton. Therapist ZJ is a physiotherapist with nearly 20 years of clinical experience in physical therapy, and Therapist XH has 3 years of clinical experience in physical therapy. All testing was performed in a quiet room to minimise potential confounding factors.

To determine inter-rater reliability, two maximum isokinetic strength tests were conducted by ZJ and XH, respectively, using identical protocols. Each participant completed two consecutive testing sessions separated by 30 min of seated rest (24). The first test was conducted by XH and the second test by ZJ, and both testers were blinded to each other's results. Before each test, the tester provided a detailed explanation of the procedure to participants. Each tester positioned the participant in the UGO exoskeleton in accordance with the standardised setup protocol. Testing began with a familiarisation trial consisting of passive hip and knee flexion−extension movements, followed by five maximal−effort trials. For the hip assessment, participants began at 60° of hip flexion and performed extension followed by flexion, repeating this sequence five times. For the knee assessment, participants began at 0° of knee extension and performed extension followed by flexion, again repeating the sequence five times. During each test, including both the familiarisation and maximal−effort trials, instructions were displayed on the UGO exoskeleton device and also provided verbally by the tester. Participants were instructed to produce force as strongly as possible. Peak torque was captured in real time by the torque sensor and immediately displayed on the tester's screen. Peak flexion and extension torques were bilaterally recorded for both the hip and knee joints. A 2-min rest period was allowed between tests of different joints. After completing the maximum isokinetic strength tests for all four muscle groups, the participant was removed from the UGO exoskeleton. Once participants had fully recovered from fatigue, the second tester (ZJ) reinstalled each participant in the UGO exoskeleton on the same day using the same preset angular velocities and ranges of motion. She then repeated the protocol in the same manner as the first tester (XH).

Previous studies have demonstrated that increasing the number of sessions within a testing protocol affects measurement reliability (25, 26). The use of two participant samples for assessing inter- and intra-tester reliability in this study was therefore a deliberate methodological choice intended to prevent confounding and ensure the validity of each estimate. Combining both assessments in a single group would have introduced significant bias. For intra-tester reliability, requiring the same tester to complete multiple setup procedures on the same participant risks carryover effects, such as recall bias, in which the tester remembers previous adjustments and unintentionally increases consistency. For inter-tester reliability, sequential assessments by different testers on a single participant could be affected by fatigue or learning effects, thereby obscuring the true extent of between-operator variability. By using separate cohorts, we isolated these sources of error, ensuring that the intra-tester results reflect the temporal consistency of a single tester's technique and the inter-tester results reflect the true variation between different operators.This approach provided independent and unbiased estimates for both critical metrics. Thus, in this study, we examined intra-tester reliability by having a single examiner (ZJ) conduct maximal isokinetic strength assessments on 20 additional participants across two testing sessions separated by a 48–72-hour interval. Identical testing protocols were implemented in both sessions to ensure consistency in the measurement procedures.

To control for potential confounding variables, the testing times and participants' previous physical activity levels were standardised to reduce the effects of diurnal variation and exercise-induced fatigue on performance outcomes. All testing sessions for each participant were conducted at approximately the same time of day to minimize the influence of diurnal variations on muscle performance. Before testing, participants were instructed to refrain from strenuous physical exercise for at least 24 h. They were also asked to avoid unusual physical activities and maintain their usual daily routines without significant deviation.

### 2.7 Outcomes

The peak torque (Nm) of the four muscle groups was measured bilaterally. For statistical analysis, the average of the three highest peak torque values obtained from the five trials was used.

Although the primary endpoints were biomechanical reliability and metabolic cost, informal observation indicated that the system was well-tolerated by all participants, and no adverse events occurred. However, structured evaluations of usability, comfort, and fatigue using standardised scales (e.g., System Usability Scale and Borg Scale) were not conducted and therefore represent a key direction for future research.

### 2.8 Statistical analysis

#### 2.8.1 Sample size calculation

Because no previous study had investigated the reliability of the UGO exoskeleton, we assumed a conservative minimal acceptable reliability (ICC = 0.65) and an excellent expected reliability (ICC = 0.90). A sample size of at least 18 participants was required to achieve 80% power at a significance level of 0.05. The sample size was estimated using an online sample size calculator (27).

#### 2.8.2 Participants

Descriptive statistics were used to analyse the demographic characteristics of the participants. Continuous data are presented as the mean ± standard deviation (SD). Categorical data are presented as percentages and frequencies. The distribution of all variables was determined using the Kolmogorov–Smirnov test.

#### 2.8.3 Reliability

Reliability analysis began with a repeated-measures analysis of variance (ANOVA), and the F-ratio was examined to detect any systematic error (28).

Inter-tester and intra-tester reliability were analysed using the ICC, standard error of measurement (SEM), and Bland–Altman plots.

##### 2.8.3.1 ICC

Relative reliability between measurements was assessed using the ICC. To minimise potential learning effects, we used ICC Model 3 for both inter-tester and intra-tester reliability analyses. Specifically, we used ICC Model 3,2 (two-way mixed effects, absolute agreement, multiple raters) to assess inter-tester reliability and ICC Model 3,1 (two-way mixed effects, absolute agreement, single rater) to assess intra-tester reliability.

The ICC values were interpreted based on a previous study (28), with values of <0.60, 0.60–0.80, and >0.80 considered to indicate poor, good, and excellent reliability, respectively. If the value exceeds 0.20, the estimate should be considered precise enough only for group-level comparisons.

##### 2.8.3.2 SEM

The SEM, an index of absolute reliability, quantifies the precision of individual scores on a test. In this study, the SEM was calculated as the square root of the within-subjects mean square error from the repeated-measures ANOVA (29, 30): SEM = $\sqrt{MSE}$, where MSE denotes the mean square error and represents the variance attributable to the random measurement error.

SEM%, the relative standard error, was calculated as SEM% = (SEM/grand mean across both tests) × 100 (31).

The MDC indicates a clinically important change. The MDC was calculated as (28, 32) MDC = 1.96 × SEM×$\sqrt{2}$ and expressed in relative terms as MDC% = (MDC/grand mean across both tests) × 100.

##### 2.8.3.3 Bland–Altman plot

To further determine differences between the tests performed either by different testers or by the same tester at different times, a Bland–Altman plot was used to compare the mean difference and the 95% limit of agreement (LOA) between the isokinetic muscle strength tests. The 95% LOA serves a function analogous to the MDC by establishing a threshold that differentiates true biological changes (such as spontaneous recovery of impairment) from measurement noise (33). A narrower 95% LOA indicates that the measurement tool is more sensitive to detect subtle biological changes occurring over time or in response to an intervention (33, 34). The mean difference was calculated as the average discrepancy between two assessments of the same individuals. The upper and lower boundaries of the 95% LOA were determined by calculating the mean difference and then adding or subtracting 1.96 × SD (35). According to Bland–Altman criteria, a 95% LOA width that is ≤20% of the grand mean indicates an acceptable level of estimated variability (30). A smaller mean difference and narrower 95% LOA suggest better agreement between the measurements.

All statistical analyses were performed using SPSS version 20.0 (IBM, Inc., Armonk, NY, USA). All tests were two-tailed, and a *p* value of <0.05 was considered statistically significant.

## 3 Results

### 3.1 Demographic characteristics

**Figure 2:**
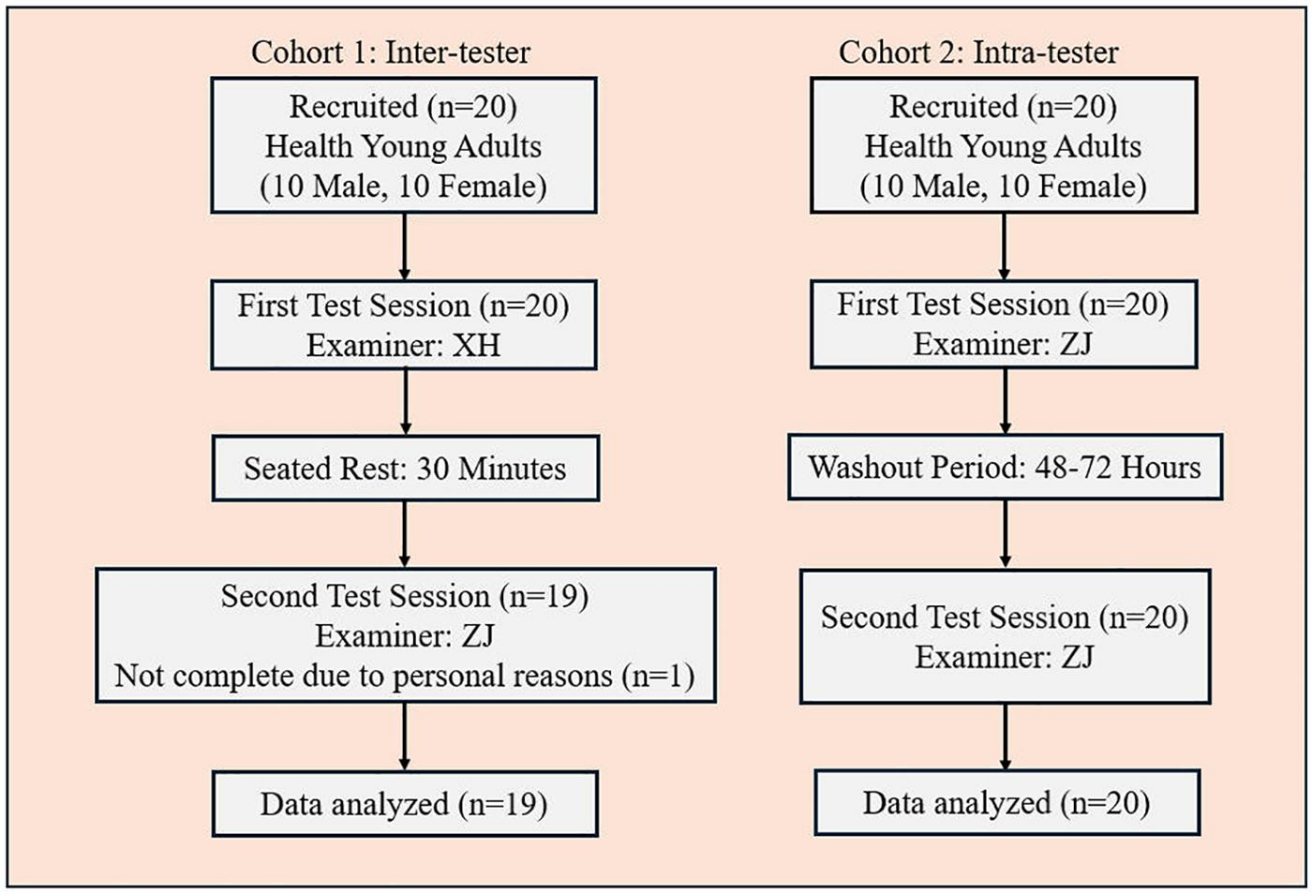
The participant flow diagram.

The participant flow diagram is illustrated in Figure 2. We enrolled two independent cohorts, each comprising 20 healthy young adults (10 men and 10 women) who were fourth-year undergraduate students completing an internship in the Department of Rehabilitation Medicine at the First Affiliated Hospital of Sun Yat-sen University. In the first cohort, one participant did not complete the second assessment after the 30-minute rest period; therefore, 19 participants completed the inter-tester evaluation. Participants in the inter-tester reliability cohort had a mean age of 22.58 ± 1.07 (range 21–25) years, whereas participants in the intra-tester reliability cohort had a mean age of 21.40 ± 0.99 (range 19–23) years. The participants' demographic characteristics are listed in Table 1.

**Table 1:** Characteristics of the study participants.

Variable	Inter-rater study Sample n = 19 Values	Intra-rater study Sample *n* = 20 Values
Sex		
Male (%)	9 (47.37)	10 (50.00)
Female (%)	10 (52.63)	10 (50.00)
Dominate side		
Right (%)	19 (100)	20 (100)
Left (%)	0 (0)	0 (0)
Age (years)	22.58 ± 1.07	21.40 ± 0.99
	(21.00–25.00)	(19.00–23.00)
Height (cm)	168.21 ± 8.32	166.45 ± 7.10
	(156.00–183.00)	(154.00–179.00)
Weight (Kg)	60.58 ± 10.45	59.30 ± 11.11
	(47.00–87.00)	(43.60–80.00)

Values were n (%) or mean ± SD (range).

### 3.2 Inter-tester reliability

The inter-tester reliability of the UGO exoskeleton's maximum isokinetic strength measurements was evaluated using data from the 19 participants who were independently assessed by ZJ and XH on the same day.

No systematic error was noted for dominant hip and knee flexors and non-dominant knee flexors; however, systematic error was observed for all other measurements (Table 2).

**Table 2:** Peak isokinetic extension and flexion torque (Nm) of the hip and knee (*n* = 19).

Variable				Test 1	Test 2	F	*P*
Hip	Right	Flexor	Total	89.488 ± 28.495	99.212 ± 28.905	4.132	0.057
			Male	107.774 ± 24.675	114.033 ± 20.091		
			Female	73.030 ± 21.151^a^	85.873 ± 29.914^a^		
		Extensor	Total	100.874 ± 31.306	119.725 ± 31.771	19.752	<0.001
			Male	121.652 ± 26.614	140.200 ± 21.764		
			Female	82.173 ± 22.588^a^	101.297 ± 28.305^a^		
	Left	Flexor	Total	97.518 ± 31.924	107.207 ± 32.230	4.519	0.048
			Male	112.967 ± 34.571	125.604 ± 27.799		
			Female	83.613 ± 22.856^a^	90.650 ± 27.341^a^		
		Extensor	Total	103.258 ± 23.033	129.768 ± 31.169	25.733	<0.001
			Male	113.144 ± 26.254	145.637 ± 28.326		
			Female	94.360 ± 16.205	115.487 ± 27.410^a^		
Knee	Right	Flexor	Total	54.386 ± 17.273	57.665 ± 18.751	1.275	0.274
			Male	64.715 ± 14.712	70.007 ± 17.371		
			Female	45.090 ± 14.199^a^	46.557 ± 12.064^a^		
		Extensor	Total	102.111 ± 35.533	119.367 ± 37.917	27.216	<0.001
			Male	123.341 ± 37.557	142.982 ± 40.316		
			Female	83.003 ± 20.374^a^	98.113 ± 19.262^a^		
	Left	Flexor	Total	83.270 ± 17.566	86.760 ± 18.584	1.595	0.223
			Male	94.751 ± 14.293	100.193 ± 16.462		
			Female	72.937 ± 13.604^a^	74.670 ± 10.345^a^		
		Extensor	Total	92.283 ± 38.326	107.690 ± 41.948	29.104	<0.001
			Male	119.137 ± 34.559	138.222 ± 39.208		
			Female	68.113 ± 22.492^a^	80.210 ± 19.534^a^		

Values are presented as the mean ± SD.

Test 1 was performed by therapist XH; Test 2 was performed by therapist ZJ. F represents the F-ratio obtained from the repeated-measures ANOVA and was used to assess systematic errors.

*P* < 0.05 indicates a significant systematic error.

a indicates a significant sex-related difference.

Reliability analysis showed that the ICC values for peak hip torque (both dominant and non-dominant limbs) ranged from 0.791 to 0.906 (*P* ≤ 0.001), indicating good-to-excellent inter-tester reliability of the UGO exoskeleton's isokinetic measurements in healthy young adults.

For hip strength assessments, the SEM values for peak torque ranged from 13.07 to 16.11 Nm (SEM% = 11.85%–15.63%), and the dominant hip extensors exhibited the smallest measurement error. The MDC values ranged from 38.94 to 44.65 Nm (MDC% = 32.85–43.32).

The maximum isokinetic knee strength measurements for both the dominant and non-dominant limbs also revealed excellent inter-tester reliability, with ICC values for peak torque ranging from 0.859 to 0.975 (all *P* < 0.001) in healthy young adults.

In the knee strength assessments, the SEM for peak torque ranged from 8.52 to 10.20 Nm (SEM% = 8.80%–15.98%). Irrespective of limb dominance, extensor measurements were consistently more precise than flexor measurements, with the non-dominant knee extensor exhibiting the lowest SEM% for peak torque. The MDC values ranged from 23.61 to 28.26 Nm (MDC% = 24.40%–44.29%).

When comparing inter-tester reliability between the hip and knee, the ICC values for peak knee torque were consistently higher, and both the SEM and SEM% were markedly lower for the knee than for the hip apart from the dominant knee flexor, which had a slightly higher SEM%.

The inter-tester reliability data for maximum isokinetic hip and knee strength assessments performed using the UGO exoskeleton are summarised in Table 3.

**Table 3:** Inter-tester reliability of peak extension and flexion torque (Nm) at the hip and knee (*n* = 19).

Variable		ICC	95%CI	*P*	SEM	SEM%	MDC	MDC%
Hip								
Right	Flexor	0.848^a^	0.605–0.941	<0.001	14.745	15.628	40.871	43.318
	Extensor	0.906^a^	0.756–0.964	<0.001	13.073	11.853	36.238	32.854
Left	Flexor	0.894^a^	0.725–0.959	<0.001	14.049	13.725	38.942	38.044
	Extensor	0.791	0.458–0.920	0.001	16.108	13.825	44.648	38.320
Knee								
Right	Flexor	0.859^a^	0.635–0.946	<0.001	8.951	15.977	24.811	44.286
	Extensor	0.960^a^	0.896–0.985	<0.001	10.195	9.207	28.260	25.519
Left	Flexor	0.875^a^	0.676–0.952	<0.001	8.516	10.017	23.605	27.765
	Extensor	0.975^a^	0.936–0.991	<0.001	8.803	8.804	24.399	24.403

ICC, intraclass correlation coefficient; CI, confidence interval; SEM, standard error of measurement; SEM%, relative standard error of measurement; MDC, minimum detectable change at a 95% CI; MDC%, relative minimum detectable change at a 95% CI.

a indicates excellent correlation.

*P* < 0.05 indicates significant reliability.

**Figure 3:**
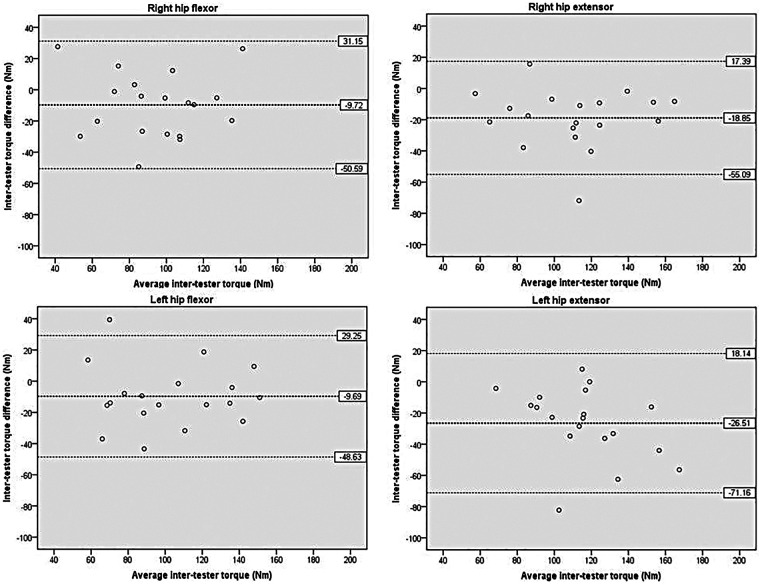
Scatterplots showing differences of hip between the two testers. The bold dashed line represents the mean difference between the testers. The dashed lines indicate the limits of agreement (mean ± 1.96 × the standard deviation of the difference scores).

Figure 3 shows the inter-tester reliability findings for hip torque. The mean difference in torque between the two testers ranged from −9.69 to −26.51 Nm, with the dominant hip flexor exhibiting the lowest value. The 95% LOA ranged from 17.39 to 31.15 Nm above the mean difference and from −48.63 to −71.16 Nm below it, with at most one outlier identified. Overall, the torque values obtained by the two testers exhibited close agreement, supported by the small mean differences and the narrow 95% LOA.

**Figure 4:**
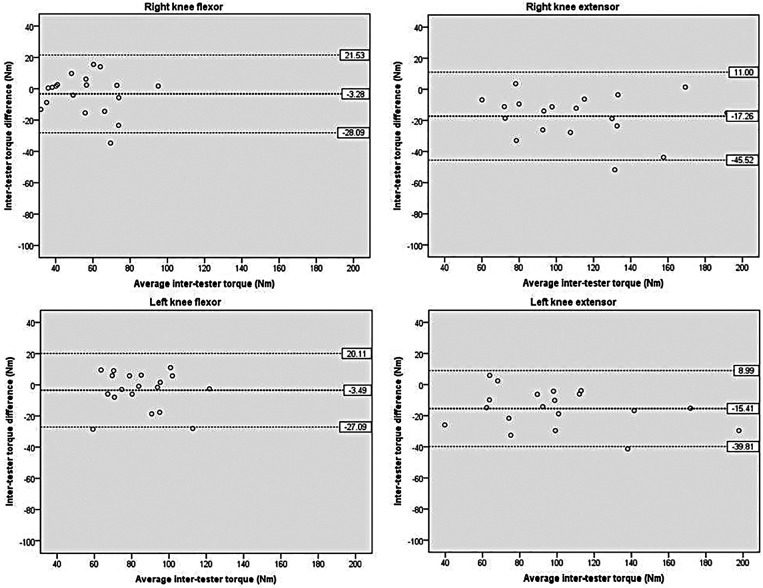
Scatterplots showing differences of knee between the two testers. The bold dashed line represents the mean difference between the testers. The dashed lines indicate the limits of agreement (mean ± 1.96 × the standard deviation of the difference scores).

Figure 4 illustrates the inter-tester reliability for knee torque. The mean difference between testers ranged from −3.28 to −17.26 Nm, with the dominant knee flexor exhibiting the lowest value. The 95% LOA ranged from −27.09 to −45.52 Nm on the lower side and from 8.99 to 21.53 Nm on the upper side, with one or two outliers noted.

### 3.3 Intra-tester reliability

The intra-tester reliability of the UGO exoskeleton's maximal isokinetic strength measurements was assessed in an additional cohort of 20 participants, each evaluated twice by ZJ with the two sessions spaced 48–72 h apart.

No systematic error was observed for any of the knee measurements or for the non-dominant hip flexor (Table 4).

**Table 4:** Peak isokinetic extension and flexion torque (Nm) of the hip and knee (*n* = 20).

Variable				Session 1	Session 2	F	*P*
Hip	Right	Flexor	Total	102.078 ± 26.510	109.907 ± 28.303	4.610	0.045
			Male	116.107 ± 25.587	126.140 ± 20.593		
			Female	88.050 ± 19.789^a^	93.673 ± 26.104^a^		
		Extensor	Total	91.815 ± 29.058	107.487 ± 22.167	14.425	0.001
			Male	111.343 ± 21.298	120.150 ± 12.970		
			Female	72.287 ± 21.943^a^	94.823 ± 22.644^a^		
	Left	Flexor	Total	104.337 ± 24.212	108.390 ± 28.027	1.379	0.255
			Male	122.113 ± 17.528	122.337 ± 16.356		
			Female	86.560 ± 15.103^a^	94.443 ± 30.961^a^		
		Extensor	Total	109.318 ± 24.063	119.388 ± 19.391	7.460	0.013
			Male	124.307 ± 16.627	128.563 ± 9.453		
			Female	94.330 ± 21.135^a^	110.213 ± 22.747^a^		
Knee	Right	Flexor	Total	68.773 ± 15.451	71.765 ± 16.938	2.214	0.153
			Male	78.410 ± 12.991	82.850 ± 9.841		
			Female	59.137 ± 11.350^a^	60.680 ± 15.354^a^		
		Extensor	Total	84.713 ± 27.788	90.025 ± 26.907	4.368	0.050
			Male	101.753 ± 24.910	106.557 ± 24.181		
			Female	67.673 ± 19.089^a^	73.493 ± 18.339^a^		
	Left	Flexor	Total	76.637 ± 15.806	78.070 ± 19.132	0.506	0.486
			Male	87.470 ± 13.840	89.070 ± 15.404		
			Female	65.803 ± 8.665^a^	67.070 ± 16.326^a^		
		Extensor	Total	88.297 ± 24.019	92.553 ± 26.235	3.067	0.096
			Male	105.453 ± 17.688	110.097 ± 20.444		
			Female	71.140 ± 15.842^a^	75.010 ± 18.739^a^		

Values are presented as the mean ± SD.

Session 1 was performed by therapist ZJ at the first session; Session 2 was performed by therapist ZJ at the second session; F represents the F-ratio obtained from the repeated-measures ANOVA and was used to assess systematic errors.

*P* < 0.05 indicates a significant systematic error.

a indicates a significant sex-related difference.

Reliability analysis revealed that the ICC values for peak hip torque (dominant and non-dominant limbs) ranged from 0.834 to 0.905 (*P* < 0.001), indicating excellent intra-tester reliability in healthy young adults.

For the hip assessments, the SEM for peak torque ranged from 11.53 to 13.05 Nm (SEM% = 10.20%–13.09%), with the non-dominant hip extensor exhibiting the smallest SEM%. The MDC values ranged from 30.26 to 36.17 Nm (MDC% = 28.26%–36.30%).

The maximum isokinetic knee strength measurements for both the dominant and non-dominant limbs also showed excellent intra-tester reliability, with ICC values for peak torque ranging from 0.917 to 0.955 (all *P* < 0.001).

In the knee strength measurements, the SEM for peak torque ranged from 6.36 to 8.04 Nm (SEM% = 8.24%–9.20%). Measurements from the non-dominant limb demonstrated consistently greater precision than those from the dominant limb in both flexor and extensor muscle groups, with the non-dominant knee flexor exhibiting the lowest SEM%. The MDC values ranged from 17.62 to 22.28 Nm (MDC% = 22.83%–25.50%).

For intra-tester reliability, peak-torque measurements at the knee consistently outperformed those at the hip: the ICC values were higher, SEM values were smaller, and SEM% was lower across all comparisons.

The intra-tester reliability data for maximum isokinetic hip and knee strength assessed using the UGO exoskeleton are summarised in Table 5.

**Table 5:** Intra-tester reliability of peak extension and flexion torque (Nm) at the hip and knee (*n* = 20).

Variable		ICC	95%CI	*P*	SEM	SEM%	MDC	MDC%
Hip								
Right	Flexor	0.903^a^	0.755–0.962	<0.001	11.530	10.878	31.959	30.152
	Extensor	0.854^a^	0.631–0.942	<0.001	13.048	13.094	36.168	36.295
Left	Flexor	0.905^a^	0.760–0.962	<0.001	10.915	10.262	30.255	28.445
	Extensor	0.834^a^	0.581–0.934	<0.001	11.659	10.196	32.317	28.261
Knee								
Right	Flexor	0.917^a^	0.789–0.967	<0.001	6.358	9.048	17.624	25.081
	Extensor	0.955^a^	0.886–0.982	<0.001	8.037	9.199	22.277	25.497
Left	Flexor	0.929^a^	0.822–0.972	<0.001	6.372	8.238	17.663	22.834
	Extensor	0.951^a^	0.876–0.981	<0.001	7.686	8.500	21.304	23.560

ICC, intraclass correlation coefficient; CI, confidence interval; SEM, standard error of measurement; SEM%, relative standard error of measurement; MDC, minimum detectable change at a 95% CI; MDC%, relative minimum detectable change at a 95% CI.

a indicates excellent correlation.

*P* < 0.05 indicates significant reliability.

**Figure 5:**
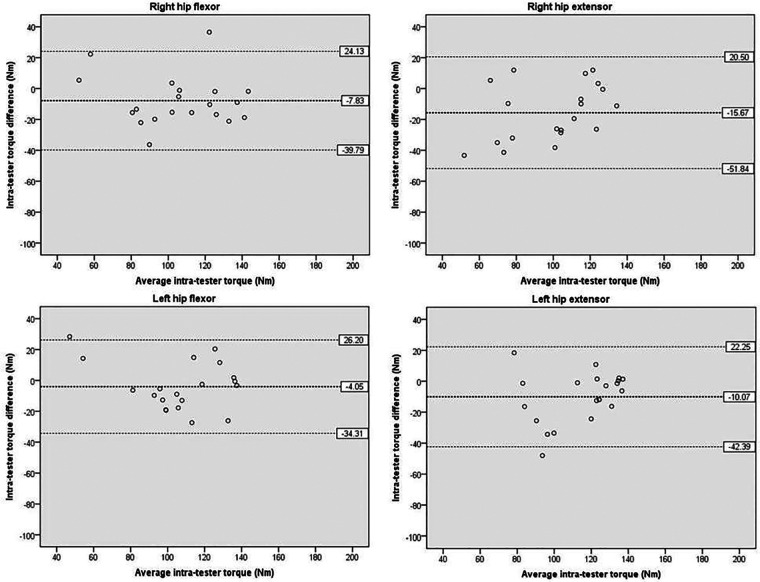
Scatterplots showing differences of hip between the two measurements performed by the same tester at different times. The bold dashed line indicates the mean difference between sessions. The dashed line indicates the limits of agreement (mean ± 1.96 × the standard deviation of the difference scores).

Figure 5 presents the intra-tester reliability analysis for hip torque. The mean difference between the two testing sessions performed by the same examiner ranged from −4.05 to −15.67 Nm, with the non-dominant hip flexor exhibiting the smallest difference. The 95% LOA ranged from −34.31 to −51.84 Nm on the lower side and from 20.50 to 26.20 Nm on the upper side, with at most one outlier observed. The small mean differences and the narrow 95% LOA together confirmed good-to-excellent agreement between sessions.

**Figure 6:**
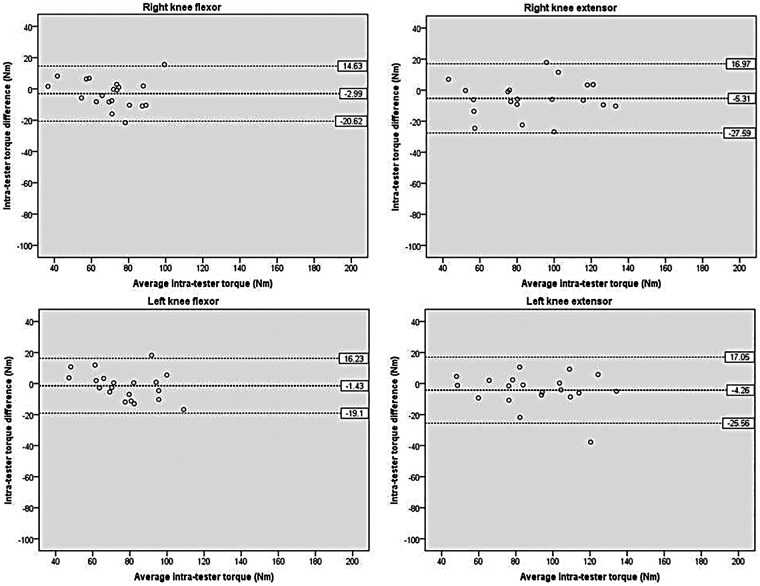
Scatterplots showing differences between the two measurements of knee by the same tester at different times. The bold dashed line indicates the mean difference between sessions. The dashed line indicates the limits of agreement (mean ± 1.96 × the standard deviation of the difference scores).

Figure 6 depicts the intra-tester reliability for knee torque. The mean difference between sessions ranged from −1.43 to −5.31 Nm, with the non-dominant knee flexor exhibiting the smallest difference. The 95% LOA ranged from −19.10 to −27.59 Nm on the lower side and from 14.63 to 17.05 Nm on the upper side, with one outlier noted. The minimal mean differences and the narrow 95% LOA together confirmed good-to-excellent consistency across sessions.

### 3.4 Sex-related differences in muscle strength

To investigate sex-related differences, a *post hoc* analysis was performed. A significant main effect of sex was observed, with independent-samples *t* tests indicating that male participants had higher peak torque values in nearly all tested muscle groups (hip and knee flexors and extensors; all *P* < 0.05). The only exception was the left hip extensor during Test 1, for which no significant difference was observed. Detailed data are presented in Tables 2, 4.

## 4 Discussion

This study examined the reliability of the UGO exoskeleton for measuring hip and knee flexor and extensor peak torque in healthy young adults. Our results demonstrated that the measurements of both hip and knee flexors and extensors demonstrated good-to-excellent inter- and intra-tester reliability. These findings indicate that this protocol can be used as an objective outcome measure in rehabilitation practice. This approach enables therapists to evaluate patients' muscle status when they are standing in the UGO exoskeleton, thereby saving time, aligning the assessment with natural walking posture, and providing a reliable method for monitoring and documenting the rehabilitation process.

Our reliability analysis revealed good-to-excellent inter-tester (ICC = 0.791–0.975) and intra-tester (ICC = 0.834–0.955) reliability for hip and knee flexor and extensor measurements obtained using the UGO exoskeleton. *These* findings are comparable to the good-to-excellent inter-tester reliability (ICC = 0.72–0.95) and intra-tester reliability (ICC = 0.71–0.90) reported in healthy adults using a driven gait orthosis (Lokomat) (36), but exceed the reliability reported for hand-held dynamometry in the same population (37). The inter-rater reliability of HHD depends mainly on the examiner's strength (38), introducing a source of variability that is removed through the UGO exoskeleton's rigid fixation. Furthermore, every participant is assessed in a strictly standardised, near-gait posture, ensuring that measurements are both consistent and ecologically valid. Our results are also better than the fair-to-excellent reliability reported for Lokomat-based assessments in adults with neuromuscular disorders (ICC = 0.66–0.97 for inter-tester reliability) (36). The wider variability observed in that population is consistent with the literature indicating that isokinetic reliability varies substantially, with ICCs ranging from 0.60 to 0.99 depending on the joint tested, angular velocity, and population characteristics (39, 40). Repeatedly producing a maximal isometric force requires high motivation and sustained concentration (41). Compared with young healthy adults, adults with neuromuscular disorders may have lower motivation and concentration, slower response times, decreased sensitivity, and poorer coordination. These factors are likely to contribute to the lower reliability observed in that population.

SEM is the most clinically relevant metric because it enables clinicians to determine whether an observed change—such as one produced by an intervention—exceeds the expected bounds of measurement error (42). The SEM% values were 10.20%–13.09% for hip intra-tester reliability and 8.24%–9.20% for knee intra-tester reliability and 13.07%–16.11% for hip inter-tester reliability and 8.52%–10.20% for knee inter-tester reliability, indicating acceptable within-subject variation (31).

The relative change values (MDC%) were 28.26%–36.30% for hip intra-tester reliability and 22.83%–25.50% for knee intra-tester reliability and 32.85%–43.32% for hip inter-tester reliability and 24.40%–44.29% for knee inter-tester reliability. Strength increases of approximately 30% to 50% have been reported in youth after short-term resistance training programs lasting 8–20 weeks (43, 44). Accordingly, the MDC% values observed in the present study are acceptable and sufficiently small to detect clinically meaningful changes.

The results of this study demonstrated that inter-tester reliability was lower than intra-tester reliability. This finding differed from that reported by Bolliger et al. (36). One possible explanation for the lower inter-tester reliability is that the exoskeleton system requires examiners to account for individual anthropometry, and this process contains a degree of subjectivity. A second possible reason is that residual neuromuscular excitation from the first testing session may have persisted and affected force output during the second session conducted later the same day. A third possible reason is a learning effect, which can be a source of systematic error. A consistent improvement in performance was observed across participants during the second measurement session. Although this is likely to reflect increased familiarity with the exoskeleton and testing protocol, it is important to acknowledge that test–retest reliability in clinical settings naturally captures these practical sources of variance. Therefore, our obtained ICC and SEM values represent realistic estimates of measurement consistency that include the effect of initial familiarisation. Future studies incorporating a third or even fourth testing session would clarify whether performance stabilises after the early learning phase and help identify the point at which a stable baseline is reached for tracking long-term rehabilitation progress. The F-ratio from the repeated-measures ANOVA supported this finding. Across the eight trials, systematic bias was detected in five inter-rater comparisons (*P* < 0.05) and in three intra-rater comparisons (*P* < 0.05). It is also plausible that shorter intervals between repeated tests magnify the learning effect. For future inter-tester reliability studies, extending the interval between sessions would be advisable to ensure the full dissipation of muscle fatigue, which recovers at markedly different rates among individuals.

Compared with knee force assessments, hip force measurements exhibited lower reliability, indicating that controlled hip flexion and extension are inherently more difficult to standardise than the analogous knee tasks. This finding aligns with the results of Meldrum *et al.* (45) and Bolliger *et al.* (36), who likewise reported poorer inter- and intra-rater reliability for hip movements than for knee movements. Several factors may explain this pattern. First, hip flexor and extensor muscles typically generate higher torque than their knee counterparts, necessitating more robust fixation. In young healthy individuals, the waist is generally narrower than the bi-trochanteric diameter; thus, the waist can be stabilised only with soft straps instead of the rigid metal supports available around the knee. This configuration permits greater movement and lower measurement reliability at the hip. Second, because the hip joint is a ball-and-socket joint with three degrees of freedom of movement and the knee joint is a trocho-ginglymus joint that mainly performs flexion and extension, achieving stable fixation for maximal hip strength testing is more difficult. Third, although the UGO exoskeleton provides sufficient fixation for gait training, its fixation around the hip is less secure than that around the knee joint when performing maximal muscle strength testing. The thigh strap is positioned distally on the thigh, whereas the calf strap is positioned proximally on the end of the calf. In addition, stability at the knee is enhanced not only by the outer metal rod but also by a hard plastic shaft positioned at the front of the lower leg. In contrast, for hip fixation, the only additional support apart from the external metal frame is a soft cushion positioned behind the buttocks, which provides little support, together with an abdominal waistband that provides the remaining support. The participants were repeatedly instructed to remain upright and fix their gaze forward to minimise compensatory movements, which might have reduced this effect. Fourth, differences in movement patterns are another probable contributor. During hip flexion and extension testing, knee flexion and extension inevitably occur alongside the intended hip movement. In contrast, during knee flexion and extension testing, the shank moves while the hip remains stationary. Compared with knee testing, the movement during hip flexion and extension strength testing more closely resembles the joint excursions used during natural gait.

Our findings further suggest that reliability was affected by test order. Testing always followed a fixed sequence—dominant hip, non-dominant hip, dominant knee, and non-dominant knee—with fixed movement patterns: extension–flexion at the hip and flexion–extension at the knee. Reliability consistently favored the later tests in the sequence. Whether inter- or intra-tester reliability was examined, ICCs were higher for the non-dominant than the dominant limb (except for the hip extensors) and higher for the knee than for the hip. Furthermore, regardless of whether the assessment involved inter- or intra-tester comparisons, peak-torque values recorded during the second test were always greater than those obtained in the first. The participants performed better in the second trial than the first trial because they benefited from the experience of the first trial and may have been motivated to improve their performance (30). However, systematic errors (e.g., learning effects and fatigue) have been described as a natural phenomenon and therefore do not contribute to unreliability in test-retest situations (46).

In addition, when compared with the findings of previous studies (47–49), reporting knee flexor/extensor muscle strength ranges of 17.3–634 Nm and 28.3–1244 Nm and hip flexor/extensor muscle strength ranges of 27.3–210 Nm and 24.6–380 Nm, respectively, the muscle strength performance observed in our study can be interpreted as lower. The substantial variability in lower-limb strength values across studies is largely attributable to methodological differences, participant characteristics, and device constraints. For instance, in the study by Stotz et al. (49), the sample comprised 18 healthy young men (age 24.8 ± 1.9 years, height 181.6 ± 7.2 cm, mass 81.2 ± 8.5 kg). Knee strength was assessed in a seated position, whereas hip strength was measured in a supine position. Pasco et al. (48) evaluated hip flexor and abductor strength using a hand-held dynamometer (reported in kilograms), with participants positioned in either seated or side-lying positions. In contrast, our study included both male and female participants and assessed muscle strength in an upright position. These methodological and demographic differences—particularly the functional demands associated with upright posture and the inclusion of a more diverse population—are likely to have contributed to the lower strength values recorded in our investigation. Our statistical findings also confirm sex-related differences in strength.

## 5 Limitations

This study has several limitations that warrant consideration. First, our findings were obtained exclusively from healthy young adults and therefore cannot be generalised to individuals with neuromuscular disorders. Second, both intra- and inter-tester performance may be influenced by how participants are positioned and secured within the UGO exoskeleton, as well as by the participant's motivation or cooperation, which can vary between days and between testers. Third, the UGO system is designed for individuals with heights between 1.5 and 1.9 m and body weights below 100 kg. Fourth, inter-tester reliability was assessed only within a single day; conducting assessments on separate days in future studies would provide more comprehensive insights into the device's reliability. Finally, in clinical settings, the UGO exoskeleton is mainly used for gait training, which limits its wider adoption as a routine clinical assessment tool.

## 6 Conclusion

In summary, our preliminary data demonstrate that the UGO exoskeleton has good-to-excellent inter- and intra-tester reliability in healthy young adults. Bland–Altman plots further confirmed good inter-tester agreement in the assessment of lower limb strength. However, the system has not yet been validated in clinical populations. Future research should therefore investigate the full psychometric properties of the UGO exoskeleton across diverse populations.

## Data Availability

The raw data supporting the conclusions of this article will be made available by the authors, without undue reservation.

## References

[1] Medeiros DG Ferreira LF Lamp J Telles DRL. The impact of resistance training in patients diagnosed with metabolic dysfunction-associated steatotic liver disease: a systematic review. Eur J Gastroen Hepat. (2025) 37:129–36. 10.1097/MEG.0000000000002887 PMC11658022 39589803

[2] Shelly S Gotkine M Wilf YA Lotan I Abraham A Dori A National guidelines for diagnosis, treatment, and management of myasthenia gravis in Israel. Ther Adv Neurol Diso. (2025) 18:51324601. 10.1177/17562864251361607 PMC12322348 40766205

[3] Habboub B Speer R Gosch M Singler K. The diagnosis and treatment of sarcopenia and sarcopenic obesity. Dtsch Arztebl Int. (2025) 122:121–6. 10.3238/arztebl.m2025.0004 39838543 PMC12452630

[4] Gaudet J Handrigan G. Assessing the validity and reliability of A low-cost microcontroller-based load cell amplifier for measuring lower limb and upper limb muscular force. Sensors-Basel. (2020) 20(17):4999. 10.3390/s20174999 32899264 PMC7506672

[5] Herbosa CG Perez R Jaeger A Dy CJ Brogan DM. Inhibition of SARM1 reduces neuropathic pain in a spared nerve injury rodent model. Muscle Nerve. (2025) 71:670–9. 10.1002/mus.28367 39936361 PMC12096083

[6] Hopper DM Goh SC Wentworth LA Chan DYK Chau JHW Wootton GJ Test–retest reliability of knee rating scales and functional hop tests one year following anterior cruciate ligament reconstruction. Phys Ther Sport. (2002) 3:10–8. 10.1054/ptsp.2001.0094

[7] Picot B Terrier R Forestier N Fourchet F McKeon PO. The star excursion balance test: an update review and practical guidelines. Int J Athletic Ther Train. (2021) 26:285–93. 10.1123/ijatt.2020-0106

[8] Li D Liu Y Feng Y Peng C Tang D. Interlimb asymmetries of lower limb isometric strength for predicting plantar fasciitis in male amateur marathon runners: a prospective cohort study. BMC Sports Sci Med R. (2025) 17:255. 10.1186/s13102-025-01295-z PMC12395752 40877885

[9] Kwakkel G Stinear C Essers B Munoz-Novoa M Branscheidt M Cabanas-Valdes R Motor rehabilitation after stroke: european stroke organisation (ESO) consensus-based definition and guiding framework. Eur Stroke J. (2023) 8:880–94. 10.1177/23969873231191304 37548025 PMC10683740

[10] Roth R Donath L Kurz E Zahner L Faude O. Absolute and relative reliability of isokinetic and isometric trunk strength testing using the IsoMed-2000 dynamometer. Phys Ther Sport. (2017) 24:26–31. 10.1016/j.ptsp.2016.11.005 27964928

[11] Thompson BJ Xu J. Isokinetic dynamometer leg extensor peak torque measurement: a time-delayed reliability and score selection analysis study. J Funct Morphol Kinesiol. (2023) 8(2):62. 10.3390/jfmk8020062 37218858 PMC10204485

[12] Thompson BJ. It’s time to re-evaluate the reporting of common measures from isokinetic dynamometers: isokinetic for torque, isotonic for power. Front Sports Act Liv. (2025) 7:1472712. 10.3389/fspor.2025.1472712 PMC11832532 39968187

[13] Santos D Bravo-Sanchez A Peyre-Tartaruga LA Simini F Zacca R. Isometric force-time curve assessment: accuracy, precision, and repeatability of a Mobile application and portable and lightweight device. Sports. (2024) 12(10):268. 10.3390/sports12100268 39453234 PMC11511426

[14] Cozette M Lepretre PM Doyle C Weissland T. Isokinetic strength ratios: conventional methods, current limits and perspectives. Front Physiol. (2019) 10:567. 10.3389/fphys.2019.00567 31164830 PMC6536638

[15] Dirnberger J Huber C Hoop D Kösters A Müller E. Reproducibility of concentric and eccentric isokinetic multi-joint leg extension measurements using the IsoMed 2000-system. Isokinet Exerc Sci. (2013) 21:195–202. 10.3233/IES-130511

[16] Yoo T Kim S Burland JP Glaviano NR. Reliability of hand-held dynamometer in measuring gluteal muscle rate of torque development and peak torque: push and pull configurations. Int J Sports Phys Th. (2025) 20:595–605. 10.26603/001c.133550 PMC11964699 40182912

[17] Martins J Da SJ Da SM Bevilaqua-Grossi D. Reliability and validity of the belt-stabilized handheld dynamometer in hip- and knee-strength tests. J Athl Training. (2017) 52:809–19. 10.4085/1062-6050-52.6.04 PMC5634229 28787180

[18] Mazzocato D Biasol V Arcuri P Fairplay T Vita F Danilo D Improving wrist strength assessment reliability: a review of handheld dynamometry protocols and their clinical implications. J Clin Med. (2025) 14(14):5059. 10.3390/jcm14145059 40725753 PMC12294855

[19] Dekkers TA Blake C Collins KD McVeigh JG Sullivan O J K. Trunk strength and endurance testing in field-athletes: a reliability study. Phys Ther Sport. (2025) 75:105–12. 10.1016/j.ptsp.2025.07.012 40819517

[20] Aerts F Sheets H Anderson C Bussie N Hoskins R Maninga A Reliability and agreement of hand-held dynamometry using three standard rater test positions. Int J Sports Phys Th. (2025) 20:243–52. 10.26603/001c.128286 PMC11788088 39906058

[21] Nasr A Hunter J Dickerson CR McPhee J. Evaluation of a machine-learning-driven active-passive upper-limb exoskeleton robot: experimental human-in-the-loop study. Wearable Technol. (2023) 4:e13. 10.1017/wtc.2023.9 38487766 PMC10936398

[22] Moscatelli N Brambilla C Lanzani V Tosatti LM Scano A. Assessing transparency of robots, exoskeletons, and assistive devices: a systematic review. Sensors-Basel. (2025) 25(14):4444. 10.3390/s25144444 40732572 PMC12298396

[23] Yang W Zhang J Zhang S Yang C. Lower limb exoskeleton gait planning based on crutch and human-machine foot combined center of pressure. Sensors-Basel. (2020) 20(24):7216. 10.3390/s20247216 33339443 PMC7766720

[24] Park DS Lee G. Validity and reliability of balance assessment software using the nintendo wii balance board: usability and validation. J Neuroeng Rehabil. (2014) 11:99. 10.1186/1743-0003-11-99 24912769 PMC4074461

[25] Thomas JM Higgs S Dourish CT. Test-retest reliability and effects of repeated testing and satiety on performance of an emotional test battery. J Clin Exp Neuropsyc. (2016) 38:416–33. 10.1080/13803395.2015.1121969 PMC4784484 26702993

[26] Grgic J Scapec B Mikulic P Pedisic Z. Test-retest reliability of isometric mid-thigh pull maximum strength assessment: a systematic review. Biol Sport. (2022) 39:407–14. 10.5114/biolsport.2022.106149 35309521 PMC8919882

[27] Walter SD Eliasziw M Donner A. Sample size and optimal designs for reliability studies. Stat Med. (1998) 17:101–10. 10.1002/(sici)1097-0258(19980115)17:1<101::aid-sim727>3.0.co;2-e 9463853

[28] Weir JP. Quantifying test-retest reliability using the intraclass correlation coefficient and the SEM. J Strength Cond Res. (2005) 19:231–40. 10.1519/15184.1 15705040

[29] Stratford PW Goldsmith CH. Use of the standard error as a reliability index of interest: an applied example using elbow flexor strength data. Phys Therapy. (1997) 77:745–50. 10.1093/ptj/77.7.745 9225846

[30] Hopkins WG. Measures of reliability in sports medicine and science. Sports Med. (2000) 30:1–15. 10.2165/00007256-200030010-00001 10907753

[31] Fagher K Fritzson A Drake AM. Test-Retest reliability of isokinetic knee strength measurements in children aged 8 to 10 years. Sports Health. (2016) 8:255–9. 10.1177/1941738116632506 26895853 PMC4981066

[32] Beckerman Vogelaar TW Lankhorst GJ Verbeek AL. A criterion for stability of the motor function of the lower extremity in stroke patients using the fugl-Meyer assessment scale. Scand J Rehabil Med. (1996) 28:3–7. 10.2340/16501977199637 8701234

[33] Bland JM Altman DG. Statistical methods for assessing agreement between two methods of clinical measurement. Lancet. (1986) 1:307–10. 10.1016/S0140-6736(86)90837-8 2868172

[34] Koo TK Li MY. A guideline of selecting and reporting intraclass correlation coefficients for reliability research. J Chiropr Med. (2016) 15:155–63. 10.1016/j.jcm.2016.02.012 27330520 PMC4913118

[35] Bland JM Altman DG. Agreement between methods of measurement with multiple observations per individual. J Biopharm Stat. (2007) 17:571–82. 10.1080/10543400701329422 17613642

[36] Bolliger M Banz R Dietz V Lunenburger L. Standardized voluntary force measurement in a lower extremity rehabilitation robot. J Neuroeng Rehabil. (2008) 5:23. 10.1186/1743-0003-5-23 18957092 PMC2596777

[37] Scott DA Bond EQ Sisto SA Nadler SF. The intra- and interrater reliability of hip muscle strength assessments using a handheld versus a portable dynamometer anchoring station. Arch Phys Med Rehab. (2004) 85:598–603. 10.1016/j.apmr.2003.07.013 15083436

[38] Wikholm JB Bohannon RW. Hand-held dynamometer measurements: tester strength makes a difference. J Orthop Sport Phys. (1991) 13:191–8. 10.2519/jospt.1991.13.4.191 18796845

[39] De Ste CM Deighan M Armstrong N. Assessment and interpretation of isokinetic muscle strength during growth and maturation. Sports Med. (2003) 33:727–43. 10.2165/00007256-200333100-00002 12895130

[40] Carvalho HM Coelho ESM Ronque ER Goncalves RS Philippaerts RM Malina RM. Assessment of reliability in isokinetic testing among adolescent basketball players. Medicina-Lithuania. (2011) 47:446–52. 10.3390/medicina47080063 22123556

[41] Wilson GJ Murphy AJ. The use of isometric tests of muscular function in athletic assessment. Sports Med. (1996) 22:19–37. 10.2165/00007256-199622010-00003 8819238

[42] de Vet HC Terwee CB Knol DL Bouter LM. When to use agreement versus reliability measures. J Clin Epidemiol. (2006) 59:1033–9. 10.1016/j.jclinepi.2005.10.015 16980142

[43] Dahab KS McCambridge TM. Strength training in children and adolescents: raising the bar for young athletes? Sports Health. (2009) 1:223–6. 10.1177/1941738109334215 23015875 PMC3445252

[44] Faigenbaum AD Kraemer WJ Blimkie CJ Jeffreys I Micheli LJ Nitka M Youth resistance training: updated position statement paper from the national strength and conditioning association. J Strength Cond Res. (2009) 23:S60–79. 10.1519/JSC.0b013e31819df407 19620931

[45] Meldrum D Cahalane E Keogan F Hardiman O. Maximum voluntary isometric contraction: investigation of reliability and learning effect. Amyotroph Lateral Scler Other Motor Neuron Disord. (2003) 4:36–44. 10.1080/14660820310006715 12745617

[46] Rousson V Gasser T Seifert B. Assessing intrarater, interrater and test-retest reliability of continuous measurements. Stat Med. (2002) 21:3431–46. 10.1002/sim.1253 12407682

[47] Schindler I Pontes SS Bertoni M Junior GF Junior B de Jesus F A systematic review of isokinetic muscle strength in a healthy population with special reference to age and gender. Sports Health. (2023) 15:328–32. 10.1177/19417381221146258 36645122 PMC10170235

[48] Pasco JA Stuart AL Holloway-Kew KL Tembo MC Sui SX Anderson KB Lower-limb muscle strength: normative data from an observational population-based study. Bmc Musculoskel Dis. (2020) 21:89. 10.1186/s12891-020-3098-7 PMC7007641 32035479

[49] Stotz A Maghames E Mason J Groll A Zech A. Maximum isometric torque at individually-adjusted joint angles exceeds eccentric and concentric torque in lower extremity joint actions. Bmc Sports Sci Med R. (2022) 14:13. 10.1186/s13102-022-00401-9 PMC8783437 35063013

